# EXPLORING THE DAY-TO-DAY DYNAMICS AMONG FLOW EXPERIENCE, SUBJECTIVE AGE, AND POSITIVE AFFECT IN THE UNITED KINGDOM

**DOI:** 10.1093/geroni/igae098.1737

**Published:** 2024-12-31

**Authors:** Dwight Tse, Samuel Finnigan

**Affiliations:** University of Strathclyde, Glasgow, Scotland, United Kingdom; University of Strathclyde, Glasgow, Scotland, United Kingdom

## Abstract

The subjective experience of aging can vary day-to-day, and can depend upon a person’s activities and experiences. Activities conducive to flow experiences (energized experiences of intense focus and enjoyment) are associated with participants feeling younger on the same day, as well as increased levels of positive affect. Younger subjective age has similarly been found to increase positive affect throughout the day. We therefore hypothesized that greater daily flow experiences would predict greater positive affect both directly and indirectly through reducing subjective age. In the UK sample, participants completed up to 14 daily diaries, including questionnaires on their flow experience in their most time-consuming activity, subjective age, and positive affect. Analysis of responses from 85 participants aged 50 to 85 (mean = 62.16) with at least 7 completed daily surveys revealed significant within-person variability ranging from 44% (flow experience) to 73% (positive affect). Multilevel mediation analysis revealed flow experience was negatively related with subjective age and positively with positive affect, where on days with greater flow participants felt younger and reported higher positive affect. There was also a within-person indirect effect of flow increasing positive affect through subjective age: on days where participants experienced more flow than usual, they indirectly experienced higher positive affect through feeling younger. These findings remained robust when controlling for stressors, uplifts, and change in positive affect from the previous day. These findings suggest engaging in flow-conducive activities may provide additional benefits for older adults by increasing positive affect, partly by making them feel younger.

